# Exploring the relations of NLR, hsCRP and MCP-1 with type 2 diabetic kidney disease: a cross-sectional study

**DOI:** 10.1038/s41598-024-53567-2

**Published:** 2024-02-08

**Authors:** Yaxuan Fang, Bin Wang, Bo Pang, Zijun Zhou, Yunze Xing, Pai Pang, Dingyuan Zheng, Gang Zhang, Bo Yang

**Affiliations:** 1 Department of Nephrology, First Teaching Hospital of Tianjin University of Traditional Chinese Medicine, Liqizhuang Street, Xiqing District, Tianjin, 300380 China; 2 Department of Nephrology, National Clinical Research Center for Chinese Medicine Acupuncture and Moxibustion, Tianjin, China; 3 Department of Endocrinology, First Teaching Hospital of Tianjin University of Traditional Chinese Medicine, Tianjin, China; 4 Department of Endocrinology, National Clinical Research Center for Chinese Medicine Acupuncture and Moxibustion, Tianjin, China; 5 Tianjin University of Traditional Chinese Medicine, Tianjin, China; 6 The Community Health Service Center of Hangzhou Road Street in Tianjin Binhai New Area, Tianjin, China

**Keywords:** Kidney diseases, Endocrine system and metabolic diseases

## Abstract

Type 2 diabetic kidney disease (T2DKD) is a common microvascular complication of type 2 diabetes mellitus (T2DM), and its incidence is significantly increasing. Microinflammation plays an important role in the development of T2DKD. Based on this, this study investigated the value of inflammatory markers including neutrophil–lymphocyte ratio (NLR), high-sensitivity C-reactive protein (hs-CRP), monocyte chemoattractant protein-1 (MCP-1) in the prediction of T2DKD. This was a cross-sectional survey study. A total of 90 patients with T2DM, who were hospitalized in the nephrology and endocrinology departments of the First Teaching Hospital of Tianjin University of Traditional Chinese Medicine from June 2021 to January 2022, were included and divided into three groups (A1, A2, A3) according to the urinary albumin-to-creatinine ratio (UACR). Observe and compare the basic information, clinical and laboratory data, and the inflammatory markers NLR, hs-CRP, MCP-1. Results revealed that high levels of NLR (*OR* = 6.562, 95% CI 2.060–20.902, *P* = 0.001) and MCP-1 (*OR* = 1.060, 95% CI 1.026–1.095, *P* < 0.001) were risk factors in the development of T2DKD. Receiver operating characteristic curve analysis showed that the area under curve of NLR and MCP-1 in diagnosing T2DKD were 0.760 (95% CI 0.6577–0.863, *P* < 0.001) and 0.862 (95% CI 0.7787–0.937, *P* < 0.001). Therefore, the inflammatory markers NLR and MCP-1 are risk factors affecting the development of T2DKD, which of clinical value may be used as novel markers of T2DKD.

## Introduction

In recent years, the development of society and economy has brought great changes to people's diet structure and lifestyle. Diabetes mellitus (DM), as a metabolic disease, has a gradually increasing incidence^1^. According to statistics, there were about 536.6 million DM patients in the world by 2021, and it is expected that by 2045, the number of DM patients worldwide will increase to 783.2 million^2^. Type 2 diabetes mellitus (T2DM) is more common in clinical practice, accounting for more than 90% of the prevalence of DM^3^. Type 2 diabetic kidney disease (T2DKD), as one of the most common microvascular complications of T2DM, is a major cause of chronic kidney disease (CKD) and end-stage renal disease (ESRD) worldwide^4^, and the prevalence of T2DKD is increasing as the continuous rise of the prevalence of T2DM^5–7^.

The early onset of T2DKD is insidious and the clinical symptoms are not obvious. Once patients have persistent albuminuria, their kidney damage has been relatively obvious and develops rapidly, and 50% of the patients may develop ESRD within 10 years^8^. This kidney damage is usually irreversible and the high daily care costs of T2DKD patients also bring some economic pressure on individual families and society^4^. Therefore, early detection of T2DKD is particularly important in patients with DM. Currently, the most effective method for the diagnosis of T2DKD is kidney biopsy. But as an invasive examination method, it carries clinical risks such as infection and bleeding, as well as some patients who are unable to undergo kidney biopsy due to physical reasons. Increased albuminuria is a common clinical manifestation and the main diagnostic basis of T2DKD. However, most patients with early stages of T2DKD do not have significant albuminuria, which makes the early detection of T2DKD difficult. Therefore, it is especially important to find convenient, feasible, sensitive, and accurate markers for early detection of T2DKD.

Current studies on the pathogenesis of T2DKD believe that it is associated with disorders of glucose and lipid metabolism, kidney hemodynamic changes, abnormal activation of the renin–angiotensin–aldosterone system, oxidative stress, inflammatory reaction, genetic predisposition factors, and et al. ^9^. The microinflammatory has attracted much scholarly attention in recent years^10^. Microinflammation, which can be distinguished from the ordinary inflammatory reaction, is clinically considered as an increase of proinflammatory cytokines and other mediators that activate the immune system in circulation. This chronic low-grade inflammation damage to the kidney is the basis for the progression of T2DKD. Neutrophil–lymphocyte ratio (NLR) is the ratio of neutrophil count to lymphocyte count in peripheral blood, therefore it is not costly to obtain and is more readily available clinically compared with other inflammatory markers^11^, and it is less affected by physiological and pathological factors and more stable than neutrophil and lymphocyte count. In addition, NLR represents two immune pathways: innate immunity (primarily caused by neutrophils) and adaptive immunity (responsible by lymphocytes)^12^. Neutrophils secrete inflammatory mediators involved in epithelial and endothelial cell damage^13^, and lymphocytes regulate inflammatory response^14^. Therefore, NLR can be considered a marker of severity in many inflammation-associated diseases^15,16^ and is more predictive than assessing either parameter independently. High-sensitivity C-reactive protein (hs-CRP), a member of the pentraxin protein family, is produced by the liver and is one of the sensitive markers of inflammatory reaction^17^. Insulin resistance can lead to increased synthesis of hs-CRP, and then activate the complement to produce a large number of inflammatory mediators and release oxygen radicals, which can impair vascular endothelial cell function and increase vascular permeability^18^, leading to albuminuria production and kidney function reduced^19^. Monocyte chemoattractant protein-1 (MCP-1) is a member of the C–C subfamily of chemokines, which can be overexpressed by high glucose to induce monocyte aggregation in kidney tissues, causing inflammatory infiltration and damage in kidney^20^. MCP-1 can also induce the expression of specific adhesion molecules and the production of cytokines such as interleukin-1 (IL-1), interleukin-6 (IL-6), and tumor necrosis factor-α (TNF-α) by activating intracellular signal transduction pathways. It is evident that MCP-1 is not only a chemotactic substance, but also a cytokine that can regulate several functional parameters of monocytes. In addition, MCP-1 is also a major histamine-releasing factor, which can promote the release of histamine by basophils and mast cells, and regulate the phagocytosis and pro-apoptosis function of monocytes and macrophages^21^. Moreover, activated macrophages secrete pro-fibrotic factors such as transforming growth factor-β1 (TGF-β1), platelet-derived growth factor (PDGF), plasminogen activator inhibitor-1 (PAI-1), matrix metalloproteinases (MMPs) and tissue inhibitor factor of metalloproteinases-1 (TIMP-1), which play an important role in interstitial fibrosis^22,23^. As for the current studies related to MCP-1 and DKD, there are more studies on urinary MCP-1 and fewer on serum MCP-1. In conclusion, our study aimed to investigate whether the inflammatory markers NLR, hs-CRP, and serum MCP-1 have clinical value for T2DKD.

## Materials and methods

### Study design and population

In this study, we used the method of a cross-sectional survey to select patients hospitalized in the nephrology and endocrinology departments of the First Teaching Hospital of Tianjin University of Traditional Chinese Medicine from June 2021 to January 2022. Among them, 90 patients met the criteria of inclusion and exclusion. This study was approved by Institutional Review Board of the First Teaching Hospital of Tianjin University of Traditional Chinese Medicine. The patients were divided into three groups according to the urinary albumin-to-creatinine ratio (UACR): normal urinary albumin group (A1), UACR < 30 mg/g; micro urinary albumin group (A2), 30 ≤ UACR ≤ 300 mg/g; macro urinary albumin group (A3), UACR > 300 mg/g.

Inclusion criteria: a clear history of T2DM; adults aged 18–80 years, both genders.

Exclusion criteria: kidney disease caused by any other causes; estimated glomerular filtration rate (eGFR) < 30 mL/min/1.73 m^2^; existing obvious infection; in the acute stage of cardiovascular or cerebrovascular diseases; with acute complications of diabetes recently (such as diabetic ketoacidosis, et al.); with malignant tumors, hematological system, and autoimmune system diseases; using glucocorticoid or immunosuppressant; pregnant or lactating women.

Basic information, clinical and laboratory data of patients were recorded, including gender, age, height, weight, duration of T2DM, systolic blood pressure (SBP), diastolic blood pressure (DBP), UACR, urinary microalbumin (UmAlb), 24-h urinary protein quantity (24hUTP), serum creatinine (Scr), blood urea nitrogen (BUN), serum uric acid (SUA), eGFR, glycosylated hemoglobin (HbA_1_c), neutrophil count and lymphocyte count.

Fasting venous blood samples were obtained and centrifuged in SIGMA 3K15 centrifuge at 1043 g for 10 min to separate serum. Measurement of hs-CRP and MCP-1 was performed by enzyme-linked immunosorbent assay (ELISA). The kit was obtained from Wuhan Huamei Biotech Co., LTD., and the testing instrument was a microplate reader (Thermo Varioskan™ LUX).

Body mass index (BMI) was calculated according to the following formula BMI = weight/(height)^2^. The eGFR was calculated according to the CKD-EPI formula^24^. The NLR value was calculated by dividing the absolute neutrophil count on the absolute lymphocyte count.

### Statistical analysis

All data were processed and analyzed using SPSS 21.0 statistical software. Continuous variables were described by $\overline{X }$±*s* or *M* (*P25*, *P75*), and one-way analysis of variance (ANOVA) test was used for comparisons between groups with normal distribution, and non-parametric tests were used for non-normal distributions. Categorical variables were described as frequencies or percentages, and the chi-square test was used for comparisons between groups. The influencing factors were analyzed by logistic regression. Receiver operating characteristic (ROC) curve and the area under curve (AUC) were used to measure the diagnostic performances. *P* < 0.05 indicates that the differences were statistically significant.

### Ethics approval and consent to participate

This study was approved by Institutional Review Board of the First Teaching Hospital of Tianjin University of Traditional Chinese Medicine (Grant numbers TYLL2021[K]035). All methods were carried out in accordance with relevant guidelines and regulations. Informed consent was obtained from all subjects and/or their legal guardian(s).

## Results

### Patients characteristics

A total of 90 patients with T2DM were included in the study, including 47 males (52.20%) and 43 females (47.80%). The patients divided into three groups according to the level of UACR, diagnosed as normal urinary albumin group (A1, *n* = 30), micro urinary albumin group (A2, *n* = 41), macro urinary albumin group (A3, *n* = 19). There were no statistically significant differences in gender, age, and BMI among the groups (*P* > 0.05). The levels of UACR, UmAlb and 24hUTP in groups A1, A2 and A3 were gradually increased, while eGFR was gradually decreased, with significant differences among the three groups (*P* < 0.05). The duration of T2DM, SBP, Scr, BUN, SUA and HbA_1_c in A2 and A3 groups were significantly higher than those in A1 (*P* < 0.05). There was no significant difference in DBP level among the three groups (*P* > 0.05) (Table 1).

**Table 1:** Baseline characteristics of patients in three groups.

Variable	All (*n* = 90)	A1 (*n* = 30)	A2 (*n* = 41)	A3 (*n* = 19)	P
Males/females	47/43	17/13	21/20	9/10	0.805
Age (years)	64.06 ± 8.70	62.17 ± 7.91	64.80 ± 9.31	65.42 ± 8.43	0.339
BMI (kg/m^2^)	26.94 ± 4.56	26.06 ± 4.79	27.09 ± 4.59	28.08 ± 4.01	0.324
Duration of T2DM (years)	14.10 ± 8.58	10.83 ± 7.18	15.73 ± 9.02^a^	15.74 ± 8.57^a^	0.036
SBP (mmHg)	141.44 ± 17.19	134.20 ± 18.45^bc^	142.34 ± 17.01^a^	150.95 ± 9.16^a^	0.003
DBP (mmHg)	83.41 ± 10.19	82.43 ± 8.94	83.95 ± 11.12	83.79 ± 10.36	0.815
UACR (mg/g)	53.23 (15.09, 243.87)	6.78 (5.30, 16.33)^bc^	83.64 (41.64, 176.61)^ac^	751.00 (455.80, 1866.59)^ab^	< 0.001
UmAlb (mg/L)	34.21 (8.23, 131.47)	5.28 (2.94, 8.24)^bc^	54.38 (24.50, 101.68)^ac^	386.42 (236.82, 1040.62)^ab^	< 0.001
24hUTP (g/24h)	0.30 (0.19, 0.67)	0.19 (0.13, 0.27)^bc^	0.30 (0.23, 0.54)^ac^	1.24 (0.87, 1.94)^ab^	< 0.001
Scr (μmol/L)	72.12 (61.98, 86.51)	63.30 (56.06, 72.31)^bc^	73.58 (64.56, 85.05)^a^	101.45 (72.18, 129.02)^a^	< 0.001
BUN (mmol/L)	5.52 (4.54, 7.38)	4.58 (4.00, 5.45)^bc^	6.04 (5.09, 7.56)^a^	7.37 (5.49, 8.06)^a^	< 0.001
SUA (μmol/L)	343.68 ± 84.60	306.69 ± 68.32^bc^	356.39 ± 94.78^a^	374.69 ± 65.10^a^	0.009
eGFR (mL/min/1.73m^2^)	80.69 ± 21.08	93.59 ± 10.81^bc^	79.76 ± 20.20^ac^	62.31 ± 21.61^ab^	< 0.001
HbA_1_c (%)	7.95 ± 1.48	7.24 ± 1.12^bc^	8.29 ± 1.49^a^	8.32 ± 1.60^a^	0.005

*BMI* Body mass index, *T2DM* type 2 diabetes mellitus, *SBP* systolic blood pressure, *DBP* diastolic blood pressure, *UACR* urinary albumin-to-creatinine ratio, *UmAlb* urinary microalbumin, *24hUTP* 24-h urinary protein quantity, *Scr* serum creatinine, *BUN* blood urea nitrogen, *SUA* serum uric acid, *eGFR* estimated glomerular filtration rate, *HbA1c* glycosylated hemoglobin.

^a^*P* < 0.05 compared with A1.

^b^*P* < 0.05 compared with A2.

^c^*P* < 0.05 compared with A3.

### Inflammatory markers

**Figure 1:**
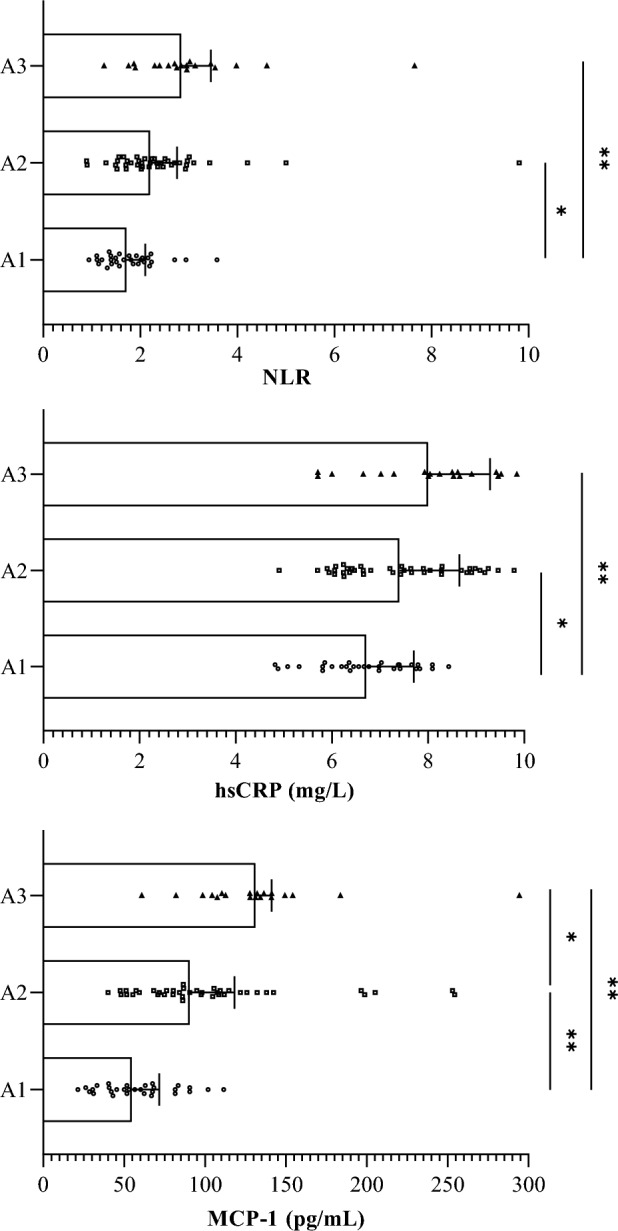
Neutrophil–lymphocyte ratio (NLR), high-sensitivity C-reactive protein (hs-CRP), and monocyte chemoattractant protein-1 (MCP-1) distribution in the study groups. *P < 0.05, **P < 0.01.

The level of MCP-1 in groups A1, A2, and A3 was gradually increased (*P* < 0.05), and the neutrophil count, NLR, and hs-CRP levels in groups A2 and A3 were significantly higher than those in groups A1 (*P* < 0.05). There was no significant difference in lymphocyte count among the three groups (*P* > 0.05) (Table 2). Graphical representations of NLR, hs-CRP, and MCP-1 distribution are shown by the bars (Fig. 1).

**Table 2:** Inflammatory markers of patients in three groups.

Variable	All (*n* = 90)	A1 (*n* = 30)	A2 (*n* = 41)	A3 (*n* = 19)	P
Neutrophil count (× 10^9^/L)	3.93 ± 1.22	3.46 ± 1.23^bc^	4.05 ± 1.18^a^	4.41 ± 1.09^a^	0.017
Lymphocyte count (× 10^9^/L)	1.84 ± 0.60	1.98 ± 0.54	1.86 ± 0.67	1.59 ± 0.46	0.087
NLR	2.09 (1.64, 2.76)	1.71 (1.38, 2.10)^bc^	2.20 (1.72, 2.76)^a^	2.84 (2.30, 3.45)^a^	< 0.001
hs-CRP(mg/L)	7.30 ± 1.26	6.71 ± 0.99^bc^	7.40 ± 1.25^a^	8.00 ± 1.29^a^	0.001
MCP-1(pg/mL)	86.15 (57.20, 116.49)	54.81 (40.66, 71.72)^bc^	90.64 (71.28, 118.29)^ac^	131.30 (107.50, 141.29)^ab^	< 0.001

^a^*P* < 0.05 compared with A1.

^b^*P* < 0.05 compared with A2.

^c^*P* < 0.05 compared with A3.

*NLR* Neutrophil–lymphocyte ratio, *hs-CRP* high-sensitivity C-reactive protein, *MCP-1* monocyte chemoattractant protein-1.

Logistic regression was conducted with the urinary albumin as the dependent variable (with = 1, without = 0) and inflammatory markers NLR, hs-CRP, and MCP-1 as the independent variables. The results showed that higher levels of NLR (*OR* = 6.562, 95% CI 2.060–20.902, *P* = 0.001) and MCP-1 (*OR* = 1.060. 95% *CI* 1.026–1.095, *P* < 0.001) were risk factors for the development of T2DKD. hs-CRP did not have a statistically significant effect on UACR (*P* > 0.05) (Table 3).

**Table 3:** The results of the multivariate logistic regression analysis.

Variable	B	S.E	Wald	P	OR	95% CI
NLR	1.881	0.591	10.128	0.001	6.562	2.060–20.902
hs-CRP	0.485	0.320	2.294	0.130	1.623	0.867–3.039
MCP-1	0.058	0.017	12.292	< 0.001	1.060	1.026–1.095

*NLR* Neutrophil–lymphocyte ratio, *hs-CRP* high-sensitivity C-reactive protein, *MCP-1* monocyte chemoattractant protein-1, *UACR* urinary albumin-to-creatinine ratio.

**Figure 2:**
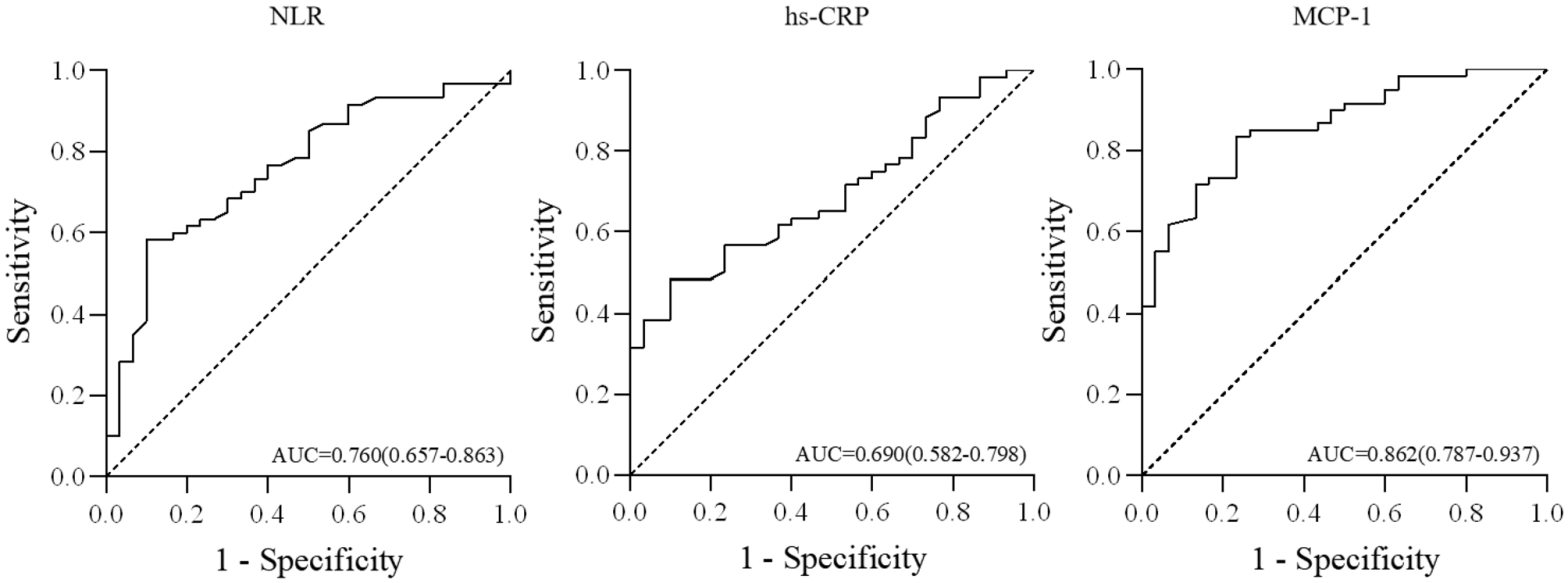
ROC curve of NLR, hs-CRP, and MCP-1 for the predictive performances in T2DKD. *ROC* Receiver operating characteristic, *AUC* area under curve, *NLR* neutrophil–lymphocyte ratio, *MCP-1* monocyte chemoattractant protein-1, *T2DKD* type 2 diabetic kidney disease.

The ROC curve analysis of NLR, hs-CRP, and MCP-1 for the development of T2DKD found the AUC of 0.760 for NLR (95% *CI* 0.657–0.863, *P* < 0.001), 0.690 for hs-CRP (95% *CI* 0.582–0.798, *P* = 0.003), and 0.862 for MCP-1 (95% *CI* 0.787–0.937,* P* < 0.001). The NLR cutoff point of 2.239 has 58.3% sensitivity and 90.0% specificity; the MCP-1 cutoff point of 69.640 pg/mL has 83.3% sensitivity and 76.7% specificity which suggest sufficient accuracy (Table 4, Fig. 2).

**Table 4:** The predictive performances of NLR, hs-CRP, and MCP-1 in T2DKD.

Variable	AUC	95%CI	Cut off	Youden index	Sensitivity	Specificity	P
NLR	0.760	0.657–0.863	2.239	0.483	0.583	0.900	< 0.001
hs-CRP	0.690	0.582–0.798	7.866 mg/L	0.383	0.483	0.900	0.003
MCP-1	0.862	0.787–0.937	69.640 pg/mL	0.600	0.833	0.767	< 0.001

*NLR* Neutrophil–lymphocyte ratio, *hs-CRP* high-sensitivity C-reactive protein, *MCP-1* monocyte chemoattractant protein-1, *UACR* urinary albumin-to-creatinine ratio, *AUC* area under curve.

## Discussion

The main objective of this study was to investigate the predictive value of inflammatory markers NLR, hs-CRP, and MCP-1 for T2DKD. The sample consisted of 90 patients with T2DM, who were divided into three groups according to UACR. The baseline characteristics and inflammatory markers were compared among the three groups. The results showed that NLR, hs-CRP, and MCP-1 were significantly correlated with UACR, and high levels of NLR and MCP-1 were risk factors affecting the development of T2DKD and had the predictive value.

NLR is considered to be an inexpensive and universally available marker of the inflammatory status of the body. Studies have shown that increased NLR is significantly associated with T2DKD and that high NLR may be a reliable predictor and a prognostic risk marker of T2DKD^25,26^. Wan et al. ^27^ also have similar results that higher NLR levels were associated with an increased prevalence of T2DKD. A three-year follow-up study showed that NLR predicted the deterioration of kidney function in patients with T2DM^28^. Zhang et al. ^29^ also suggested that increased NLR affects kidney function and histological lesions in patients with T2DM and may be an important factor in the progression of T2DKD. In our study, NLR was a risk factor for the development of T2DKD (*OR* = 6.562, 95% *CI* 2.060–20.902, *P* = 0.001). It had predictive and prognostic value for the development of T2DKD (AUC = 0.760).

Tang et al. ^30^ demonstrated that hs-CRP levels were positively correlated with the incidence of T2DKD, which may provide predictive and diagnostic values in clinical practice. A meta-analysis by Liu et al. ^31^ also had similar results, and reducing serum hs-CRP levels is beneficial for improving patients’ kidney outcomes^32^. But our study showed that there was no statistically significant between hs-CRP and UACR (*OR* = 1.623, 95% CI 0.867–3.039, *P* = 0.13). In agreement with this result, Guo et al. ^33^ found that hs-CRP was not an influential factor in the occurrence and progression of T2DKD. Therefore, it remains controversial whether hs-CRP can be an independent risk factor for T2DKD. In our study, considering the limited number and source of samples, large sample multicenter studies are necessary to investigate whether hs-CRP can be a marker for T2DKD.

Satirapoj et al. ^34^ concluded that urinary MCP-1 was an independent predictor of rapid eGFR decline and deterioration of kidney function. Shoukry et al. ^35^ and Shaker et al. ^36^ suggested that urinary MCP-1 could be a novel potential biomarker for the early diagnosis and progression of DKD. Scurt et al. ^37^ suggested that both serum and urine MCP-1 are markers and possibly mediators of early DKD and that the risk associated with serum MCP-1 is stronger. Moreover, our study revealed MCP-1 was a risk factor for the development of T2DKD (*OR* = 1.060, 95% CI 1.026–1.095, *P* < 0.001). It had the predictive and prognostic value for the development of T2DKD (AUC = 0.862) (Table 5).

**Table 5:** The study of NLR, hs-CRP, and MCP-1 in DKD.

Study	Objects	Results
Jaaban et al. (2021)^25^	158 patients with T2DM	High NLR may be served as a predictor and a prognostic risk marker of DKD
Huang et al. (2015) ^26^	253 patients with T2DM and 210 healthy subjects	High NLR values may be a reliable predictive marker of early-stage DKD
Wan et al. (2020)^27^	4797 diabetic adults in China	A higher NLR level was associated with an increased prevalence of DKD
Azab et al. (2012) ^28^	338 diabetic patients	NLR predicted the worsening of the renal function in diabetic patients
Zhang et al. (2019)^29^	247 patients with T2DM and biopsy-proven DKD	Increased NLR affects renal function and histologic lesions in patients with T2DM and may be an important factor for the progression of DKD
Tang et al. (2022)^30^	927 patients with T2DM	Hs-CRP levels were significantly and positively correlated with the presence of DKD, which may provide predictive and diagnostic values in clinical practice
Liu et al. (2015)^31^	A meta-analysis	Hs-CRP concentration can be an indicator of DKD in DM patients
Liu et al. (2020)^32^	3924 patients with IFG or diabetes	Reduction in serum hs-CRP levels favors kidney outcomes in patients with impaired fasting glucose or diabetes
Guo et al. (2020)^33^	1210 patients with T2DM	Patients with DKD had higher hsCRP levels than those without DKD, but hsCRP was not an independent risk factor for DKD after adjustment for confounders
Satirapoj et al. (2018)^34^	83 T2DM with DKD patients	Urinary MCP-1 was associated with rapid renal progression independent from conventional risk factors in DKD
Shoukry et al. (2015)^35^	75 T2DM patients and 25 healthy controls	Urinary MCP-1 may be considered as novel potential diagnostic biomarkers for the early detection of DKD
Shaker et al. (2013)^36^	60 patients with T2DM and 20 healthy volunteers	Urinary MCP-1 can be used as the markers for detection of progression of DKD. Antagonizing MCP-1 might be helpful in attenuating the progression of nephropathy in diabetic patients
Scurt et al. (2022)^37^	360 patients with T2DM	MCP-1 is a marker and possibly a mediator of early DKD

*T2DM* type 2 diabetes mellitus, *DKD* diabetic kidney disease, *NLR* Neutrophil–lymphocyte ratio, *hs-CRP* high-sensitivity C-reactive protein, *IFG* impaired fasting glucose, *MCP-1* monocyte chemoattractant protein-1.

Our study also revealed that the increase in Scr, BUN, and SUA levels and decrease in eGFR levels were parallel to albuminuria, this may predict a significant impairment of kidney function. Hypertension and hyperglycemia are also risk factors for the development of DKD^38^. The combined effect of hypertension and hyperglycemia can directly damage the microvasculature, leading to microvascular dysfunction and greatly increasing the risk of microvascular complications such as T2DKD^39^. In this study, the levels of SBP and HbA_1_c in T2DM patients with albuminuria were significantly higher than those with normal albuminuria, which can support the above view.

In addition, Corriere et al. ^40^ found that NLR can also predict the presence of other vascular diseases such as carotid plaques. The data in this part of our study is incomplete, and we will focus on this in subsequent studies. Moreover, our study is a single-center study with some limitations. The limited sample size for inclusion in observations due to time and geographical constraints. Therefore, large-sample multicenter studies are needed to verify the study results in the future.

## Conclusion

The inflammatory markers NLR and MCP-1 are risk factors affecting the development of T2DKD, which of clinical value may be used as novel predictive makers of T2DKD. However, large-sample multicenter studies are still required with follow-up to confirm their effectiveness.

## Data Availability

The data used to support the findings of this study are available from the corresponding author upon request.
